# Hypothermia in bleeding trauma: a friend or a foe?

**DOI:** 10.1186/1757-7241-17-65

**Published:** 2009-12-23

**Authors:** Tareq Kheirbek, Ashley R Kochanek, Hasan B Alam

**Affiliations:** 1 Department of Surgery, Division of Trauma, Emergency Surgery and Surgical Critical Care, Massachusetts General Hospital/Harvard Medical School, Boston, MA, USA; 2 Department of Surgery, Washington Hospital Center, Washington, DC, USA

## Abstract

The induction of hypothermia for cellular protection is well established in several clinical settings. Its role in trauma patients, however, is controversial. This review discusses the benefits and complications of induced hypothermia--emphasizing the current state of knowledge and potential applications in bleeding patients. Extensive pre-clinical data suggest that in advanced stages of shock, rapid cooling can protect cells during ischemia and reperfusion, decrease organ damage, and improve survival. Yet hypothermia is a double edged sword; unless carefully managed, its induction can be associated with a number of complications. Appropriate patient selection requires a thorough understanding of the pre-clinical literature. Clinicians must also appreciate the enormous influence that temperature modulation exerts on various cellular mechanisms. This manuscript aims to provide a balanced view of the published literature on this topic. While many of the advantageous molecular and physiological effects of induced hypothermia have been outlined in animal models, rigorous clinical investigations are needed to translate these promising findings into clinical practice.

## Introduction

Uncontrolled hemorrhage is characterized by progression from regional hypoperfusion to a state of total body ischemic insult and ends in irreversible tissue damage and death. While tissue hypoxia and excessive bleeding are undisputed harbingers of death[1], hypothermia's role in trauma is complex and context dependent. A spontaneous decrease in core body temperature following injuries correlates with poor prognosis. However, numerous studies have shown that induction of hypothermia is a potent strategy for preserving tissues and improving survival following ischemia-reperfusion events [2,3]. These studies beg the question- *is hypothermia friend or foe in trauma*?

There is no strictly accepted nomenclature to define the depth of clinically induced therapeutic hypothermia. Since the physiologic response of tissues to hypothermia differs according to its degree, it is important to stratify the current literature correspondingly. For the purposes of this review the depth of therapeutic hypothermia will be classified into: mild (33-36°C), moderate (28-32°C), deep (16-27°C), profound (6-15°C), and ultra-profound (<5°C) hypothermia. In addition, the effects of hypothermia on tissues are also modulated by numerous other variables such as timing (before or after the ischemic insult), etiology (spontaneous or induced), presence or absence of associated injuries, rate of induction and reversal, duration of hypothermia, and therapeutic approach (mild to moderate hypothermia during shock vs. profound hypothermia during arrest), to name a few. It is clearly beyond the scope of this review to categorize and analyze the literature according to all of these variables. The goal of this paper, therefore, is to provide a broad over view of the topic for the general readership, while citing the specific references in the bibliography for those who might be interested in obtaining more detailed information.

## Background

Trauma is the major cause of death in individuals aged 1 to 34 years and is the fifth leading cause of mortality in the United States--with uncontrolled hemorrhage representing the major cause of preventable deaths [4]. In spite of progress made in all major disease categories in the United States and Europe, injuries remain at the same absolute levels, and represent an increasing relative share as a major cause of mortality, especially in males [5]. The estimated cost of injuries in the U.S. was $406 billion for the year 2000 [6], which is incontrovertibly compounded by the emotional and financial burden on the patients and their families.

Traditionally, the first line approach to the bleeding trauma patients was based on replacement of acute blood losses in an attempt to maintain tissue perfusion and sustain aerobic metabolism. This therapy alone is inadequate, impractical and even harmful in many circumstances. Not only are appropriate blood products often unavailable but aggressive fluid resuscitation can actually exacerbate bleeding and worsen cellular injury [7-9]. The definitive therapy for bleeding patients continues to be surgical control of the bleeding site [10], yet most deaths from bleeding occur prior to arrival in a hospital [11], or soon after admission [12]. Furthermore, critical warm ischemia time is under five minutes for the brain [13,14] and around twenty minutes for the heart [15]. Thus, even if source of hemorrhage is controlled and spontaneous circulation restored, significant morbidity and mortality can result from the ischemia and reperfusion insults.

Dr R. Adams Cowley recognized this need for urgent care following trauma and popularized the concept of the "*Golden Hour" *in trauma care. This delineates the time during which proper management can save life but beyond which irreversible damage occurs. This principle was later adopted by the American College of Surgeons in Advanced Trauma Life Support (ATLS) training [16]. Today, the need to lengthen the Golden Hour, especially on the battlefield, remains pressing and has inspired new urgency in the development and implementation of novel therapies to protect the vital organs from hypoxic-ischemic insult.

### Pathophysiology of Hemorrhagic Shock

To understand the potential applications of hypothermia in bleeding trauma patients one must appreciate the physiologic and molecular consequences of hypovolemic hypoxic shock.

The biological response to hemorrhage is a combination of cardiovascular compensation and competing activation of cellular survival and death pathways within vital organs, especially the heart, lung, liver, kidney and brain. Physiologically, increased heart rate and catecholamines release increases the peripheral vascular resistance in order to maintain cardiac output and meet metabolic demands. Hypovolumia activates the renin-angiotensin system to retain water and sodium. However, when uncontrolled blood loss continues these mechanisms eventually fail. Tissue hypoperfusion ensues and the patient enters a state of shock.

At a cellular level, aerobic metabolism of oxidative fuels provides the energy to carry out the necessary functions. Metabolic homeostasis requires steady oxygen delivery to maintain oxidative phosphorylation in the mitochondria and generate adenosine triphosphate (ATP). Since little oxygen is stored in tissues, aerobic ATP synthesis is quickly impaired by a mismatch between oxygen supply and demand [17]_. _Significant blood loss decreases oxygen delivery (DO_2_) while oxygen uptake (VO_2_) remains fairly constant. Oxygen extraction increases to compensate for this mismatch but soon reaches its physiological limits. Aerobic VO_2 _can no longer be maintained and tissues resort to anaerobic metabolism [18,19]. In this inefficient, hypoxic and hypovolemic state, ATP utilization exceeds ATP production. Rapid depletion of intracellular ATP leads to cellular dysfunction and organ decompensation [20-23].

Notably, cells sense low oxygen tension well before ATP pools are depleted [24]. For example, hypoxic stimulus leads to increased stability and expression of hypoxia-induced factor 1 (HIF-1) in these cells. HIF-1 is a regulator for multiple molecules and pathways, such as erthropoiesis, angiogenesis, vasodilatation, and anaerobic metabolism [25]. It also activates inflammatory pathways including stress-activated protein c-jun kinase (JNK) [26] and induced nitric oxide synthase (iNOS) [27]. Nitric oxide (NO), in turn, activates nuclear factor kappa B (NF-kB), increases levels of downstream inflammatory cytokines [28] and contributes to lung and liver injury. Thus, tissues are primed for inflammatory and immune-mediated injury during this early hypoperfused, hypoxic state.

As hemorrhage progresses, hypoperfusion decouples the redox state in affected cells and initiates several unfavorable events [29]. Energy deficits secondary to shock significantly disrupt transmembrane ion transport. Intracellular Ca^+2 ^increases to deleterious levels as ATP dependent ion pumps fail to pump Ca^2+ ^out. An increased intracellular and intra-mitochondrial Ca^2+ ^concentration reduces the efficiency of oxidative phosphorylation and activates cell death pathways [30,31]. Moreover, this Ca^2+ ^excess persists and even worsens when circulation is restored.

Ineffective oxidative metabolism also leads to an accumulation of nicotinamide adenine dinucleotide (NADH), which alters pyruvate metabolism and accelerates lactate production [20]. This development correlates with an increase in morbidity and mortality following shock [32]. Likewise, supplementing resuscitation fluids with ATP-MgCl_2 _to treat this energy deficient and acidosis have been shown to restore membrane integrity [33] and mitochondrial function [34,35], attenuate lactate levels, prevent reperfusion injury to the kidney and the liver [36-41], and improve survival [42].

### Reperfusion Injury

Treating hemorrhage is more complicated than simply stopping the bleeding, replacing volume, and restoring energy losses. Aggressive fluid resuscitation is associated with cardiac and pulmonary consequences as well as coagulation disturbances, inflammation, and immunologic dysfunction [43]. The inflammation/cell damage that starts early in hemorrhage is often exacerbated by restoring the blood flow [44]. Reperfusion creates a rapid influx of oxygen and substrates for aerobic metabolic that overwhelms injured mitochondria and promotes the generation of reactive oxygen species. Restoring flow also activates neutrophils and enables them to invade affected tissues [45,46]. Invading neutrophils release inflammatory cytokines including interleukin (IL)-6, granulocyte colony-stimulating factor (G-CSF), and tumor necrosis factor alpha (TNF-alpha) [47,48]. These cytokines play an important role in perpetuating the vicious cycle of inflammation. Activation of signal transducers and activators of transcription (STAT) proteins perpetuates the inflammatory response and neutrophil invasion [49]. Activated cytokines also promote the expression of mediators in apoptotic pathways [50] that can contribute to organ dysfunction [51,52].

The sum of hemorrhage, ischemic injury and reperfusion insult manifests locally as increased cell death and systemically as organ failure. Apoptosis, necrosis, and inflammation are evident in different organs within hours after shock and resuscitation [53-56]. These early inflammatory and apoptotic changes not only indicate ischemic injury but also are precursors of post-traumatic immunosuppresion [57], acute respiratory distress syndrome (ARDS), and multiple organ failure syndrome (MODS) [58].

### Hypothermia and Hemorrhage: A History

Humans have long admired the protective properties of hypothermia, and a brief historical description might be helpful before critically analyzing the contemporary data. Historical evidence suggests that ancient cultures in Egypt and Peru performed mummification in cold chambers in the hopes of restoring their rulers to life. Inspired by observations of hibernating animals and individuals surviving extremely cold conditions, their reasoning may possess some validity, even if their methods were less than scientific.

The concept of hypothermic therapy as a remedy for febrile illness and pain relief originated in ancient medicine [59]. However, the first deliberate induction of therapeutic hypothermia did not take place until the 20^th ^century. In 1938 Temple Fay and Lawrence Smith attempted deep cooling treatments to decelerate tumor growth in cancer patients without success [60]. Over the next two decades the scientific community developed a better understanding of the physiologic and therapeutic properties of temperature modulation [61,62]. During this period, confidence in the benefits of hypothermia--its ability to decrease metabolic activity, reduce oxygen demands, and preserve tissues during ischemia--slowly expanded, along with greater insights into therapeutic cooling's risks [63].

Shortly thereafter, medical experiences in World War II and the Korean War heightened the medical and military community's awareness of hemorrhagic shock and the need for superior treatment strategies. This prompted some physicians to reevaluate the feasibility of inducing moderate (30°C) hypothermia as a treatment for severe blood loss in animals, and even try it on some patients in the battlefield [64]. These early experiments suffered from serious flaws in study designs, and not surprisingly produced conflicting results [65].

Thus, while protective hypothermia became well-established in cardiac, vascular, and neurosurgery [66,67] and is now considered beneficial in neonatal hypoxic-ischemic encephalopathy and out-of-hospital cardiac arrest [68,69], its role in traumatic injury has remained controversial.

Indeed, bleeding trauma patients differ from the elective surgical patients. In elective surgery patients, hypothermia is induced prior to ischemia under controlled settings. Even in the context of an out of hospital cardiac arrest, hypothermia is induced in patients with no additional injuries or significant blood loss. Contrastingly, trauma patients have well established tissue ischemic before they can be become candidates for induction of hypothermia. In addition, they are hemodynamically unstable, coagulopathic, and suffering from multiple wounds. Clearly, the risks and benefit of therapeutic hypothermia must be carefully weighed in these challenging patients.

Fortunately, recent studies employing more rigorous models have validated hypothermia's ability to improve survival, cellular viability, and cognitive function during hemorrhagic shock and prolonged periods of ischemia [70-74]. These findings have piqued renewed interest in therapeutic applications for trauma patients.

### Induced Therapeutic Hypothermia and Spontaneous Hypothermia: Friend and Foe

Induced hypothermia and hypothermia secondary to hemorrhagic shock are two very different physiological states that yield correspondingly disparate outcomes [75,76].

#### Spontaneous Hypothermia

Spontaneous hypothermia after major trauma is associated with greater transfusion and fluid requirements and worse outcomes [75,77,78]. Many severely injured patients arrive in the emergency department (ED) hypothermic and the incidence is increased by maneuvers performed in the ED such as clothing removal, cold fluid administration, opening body cavities, and the use of anesthetic agents [79]. Spontaneous hypothermia indicates depleted energy stores, disrupted cellular homeostasis [80,81] and therefore correlates with more severe injuries. Indeed, spontaneous hypothermia along with coagulopathy and acidosis are widely recognized as a "lethal triad" that correlates with poor outcome in trauma patients. Hypothermia (body temperature <35°C) upon admission independently associated with increased mortality in two retrospective studies that controlled for injury severity [75,81]. Yet association does not necessarily imply causality, and deliberately induced hypothermia can be life saving.

At a cellular level, ATP depletion plays a major role in the pathophysiology of spontaneous hypothermia. Since heat production in the body comes from hydrolysis of ATP to adenosine diphosphate (ADP) [82], anaerobic metabolism due to shock usually involves decreased ATP synthesis and eventually hypothermia. Seekamp et al. compared ATP levels in trauma patients (normothermic and hypothermic) to the levels in patients undergoing elective surgery (including hypothermia for coronary artery bypass operations) [83], and demonstrated that ATP levels were the lowest in trauma patients who presented with core temperature <34 degrees. Contrastingly, in the hypothermic elective surgery group, patients experienced only a small and transient decrease in ATP levels, which returned to baseline within 24 hours. Changes in ATP also correlated inversely with lactate levels - corroborating hypothermia as a sequela of energy depletion secondary to hypoxia and anaerobic metabolism. It should also be pointed out that thermoregulatory mechanisms stimulate strong sympathetic response and shivering to counteract decreasing core body temperature [84]. The resultant increase in muscular metabolic demand provokes a decompensated thermostasis and a worsened metabolic acidosis [85].

Clinically, accidental hypothermia is also associated with myocardial dysfunction, which manifest as decreased cardiac output, bradycardia, and pathognomonic J wave on the electrocardiograph. Arterial and ventricular fibrillation are subsequently seen with worsening hypothermia and central respiratory depression [86]. In a trauma/hemorrhage model, animals that became spontaneously hypothermic and were maintained at hypothermic body temperature (32°C) during resuscitation demonstrated depressed cardiac function whereas animals that were restored to normothermia during resuscitation demonstrated increased cardiac output comparable to sham animals [87].

**Figure 1:**
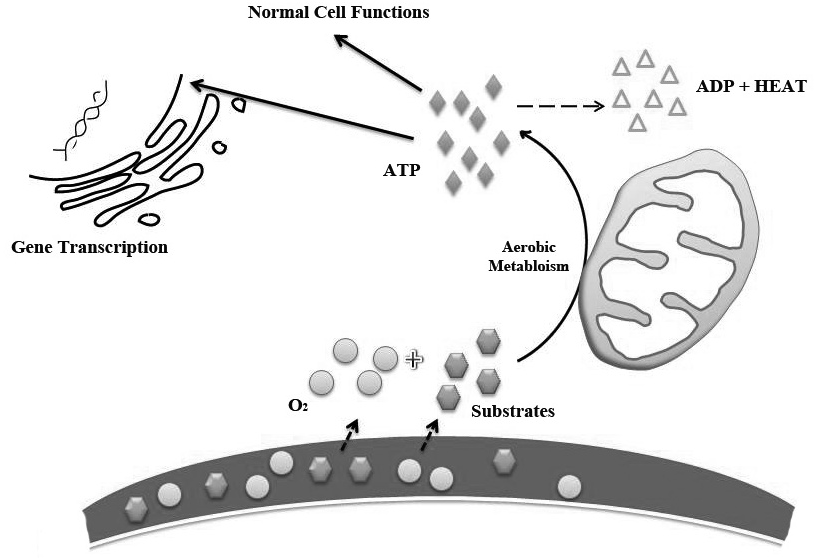
**Aerobic metabolism in the cell guarantees adequate ATP for normal physiologic function and heat production (thermoregulation)**. ATP = Adenosine Tri-Phosphate. ADP = Adenosine Di-Phosphate.

**Figure 2:**
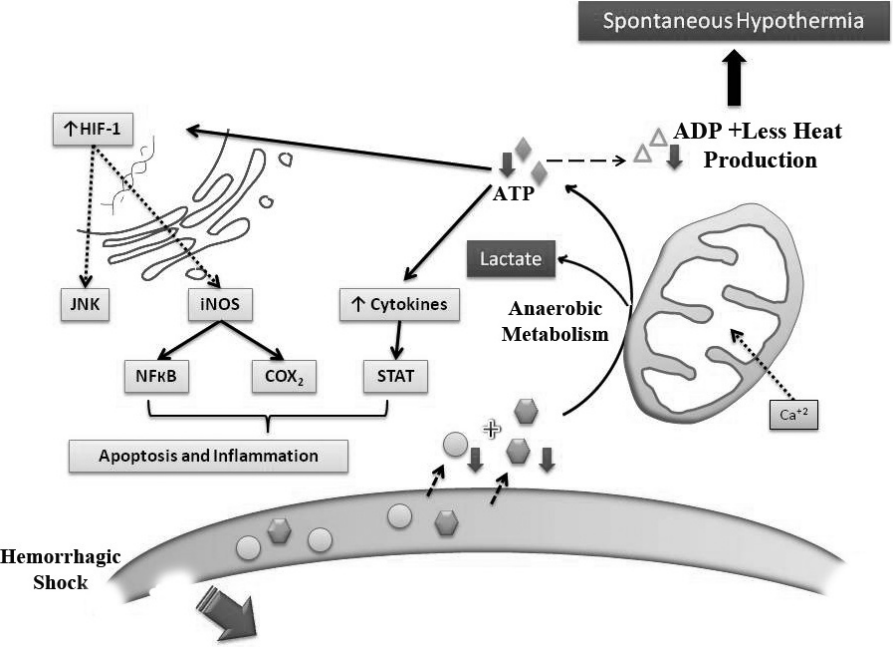
**Hemorrhage reduces availability of oxygen and substrates and stimulates a switch to anaerobic metabolism leading to decreased ATP synthesis and subsequent heat production**. This cellular hypoxia also stabilizes HIF-1, which activates several inflammatory and apoptotic pathways leading to increased cell injury and death. ATP = Adenosine Tri-Phosphate. ADP = Adenosine Di-Phosphate. HIF-1 = Hypoxia-Induced Factor-1. iNOS- induced Nitric Oxide Synthase. NO = Nitric Oxide. PG = Prostaglandin. NF-kB = Nuclear Factor- kappa B. COX-2 = cyclo-oxygenase-2. IL = Interleukins. TNFα = Tumor Necrosis Factor alpha. G-CSF = Granulocytes Colony Stimulating Factor. STAT = Signal Transducers and Activators of Transcription.

Hemodynamically, hemorrhage increases the viscosity of blood and decreases platelet and coagulation enzymes activity, worsening trauma-induced coagulopathy [88,89]. When shock is complicated by hypothermia, these deleterious changes become additive and a worse prognosis ensues [90]. Figure 1 represents a simple design of heat generation process in aerobic environment. Figure 2 illustrates the cellular changes associated with hemorrhagic shock and the development of spontaneous hypothermia.

#### Therapeutic Hypothermia

In contrast, induction of hypothermia in a controlled manner can protect tissues from ischemic injury [91,92]. As mentioned before, it requires appropriate sedation and neuromuscular blockade to minimize the deleterious effects of intense shivering that occur between 34-36°C. In this therapeutic context, controlled decreases in core body temperature are associated with decreased metabolic and oxygen demands in tissues [3], reduced activation of cell death pathways, and blunted inflammatory and immune response.

Induced hypothermia generates a state of metabolic depression that preserves cellular energy when oxygen and substrates are in limited supply. Hypothermia attenuates the activity of Na+/K+ ATPase pump that is responsible for up to 40% of ATP utilization in different tissues [93]. Cellular ATP levels decrease during shock according to the duration of ischemia. If blood flow is restored within the safe period of ischemia, ATP is regenerated [94], cellular function remains viable, and survival improves. Hypothermia can prolong this safe ischemic window. Animal studies from transplant, trauma, and brain injury literature have successfully demonstrated preservation of ATP levels in organs stored or treated with hypothermia [95,96]. Meyer et al. studied the effect of moderate, induced hypothermia after hemorrhagic shock in dogs and found decreased metabolic needs, maintained myocardial contractility and lower oxygen consumption and extraction compared to normothermic animals [97]. Ibayashi et al. showed that lowering brain temperature reduced global and regional cerebral blood flow; the concentration of brain ATP was significantly higher in hypothermic rats compared to normothermic group after 60 minutes of ischemia [98]. It is hypothesized that selective brain hypothermia decreases the basal metabolic rate in the brain, slows glucose and phsophocreatine breakdown, reduces lactate and inorganic phosphate formation, and thereby limits cellular damage during ischemia and reperfusion [99].

Induced hypothermia also alters cell survival and stress pathways that maintain tissue viability. In a rabbit model of ischemia-reperfusion, hypothermic animals exhibited decreased expression of pro-apoptotic proteins, transformation-related protein (*p53*) and *bak*, and an increased expression of anti-apoptotic Bcl-2 homologue *Bcl-x *[100]. In rodent model of hemorrhagic shock, profound hypothermia preserved Akt in cardiac myocytes, activated pro-survival proteins including Bcl-2 and beta catenin and suppressed Bad and caspase-3 activation [101]. Hypothermia also suppresses apoptosis by down regulating expression of TNF receptor (TNFR1) [102] and its associated pro-death ligand, apoptosis stimulating fragment (Fas) protein. Moderate hypothermia has been shown to suppress Fas-mediated apoptosis in stored cultured hepatocytes, along with suppression of cytochrome-c release from mitochondria and downstream activation of caspase-7 and caspase-9 [103]. Moderate hypothermia abolished hepatic STAT activity in an intestinal ischemia-reperfusion rat model, suggesting a hepatoprotective role for induced hypothermia as well [104].

**Figure 3:**
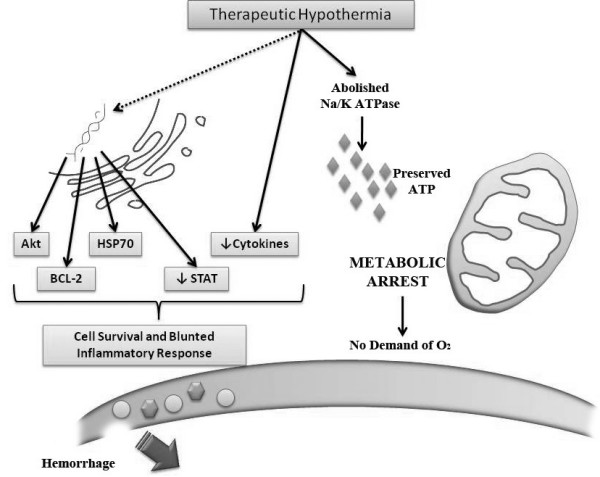
**The molecular changes associated with induced therapeutic hypothermia**. Minimal metabolic activity due to inactivation of Na^+^/K^+ ^ATPase pump conserves ATP in the cell and abolishes the need for oxygen and substrates during the circulatory arrest. Induced hypothermia also affects several molecular pathways altering the expression of many intermediates and leads to decreased inflammation, decreased neutrophils migration and decrease apoptosis. Production of ROS is blunted as well. TNFR = Tumor Necrosis Factor Receptor. Cyt-C = Cytochrome C. ROS = Reactive Oxygen Species. STAT = Signal Transducers and Activators of Transcription. HSP 70 = Heat Shock Protein. JNK = stress-activated protein c-jun Kinase.

Moreover, hypothermia significantly attenuates the inflammatory and immune response associated with hemorrhagic shock, ischemia, and surgery. In a hepatic ischemic-reperfusion model, topical deep hypothermia protected the liver from necrosis, decreased neutrophil infiltration, reduced serum levels of TNF-α, and reduced the associated lung injury [105]. In addition to modulating local immune cell invasion, induced hypothermia can decrease the release of pro-inflammatory cytokines believed to influence distant organ damage, systemic shock, and development of immune mediated pathologies such as ARDS and sepsis [106]. For example, in cardiac surgery, moderate hypothermia is associated with decreased TNF-α, IL-1, and IL-6, reduced complement activation and C-reactive protein (CRP) concentration, and an increase in anti-inflammatory IL-10 [107-110]. Figure 3 demonstrates some of the molecular changes associated with induced hypothermia

### Side Effects of Clinically Induced Hypothermia

Even though many of physiological and molecular changes in response to hypothermia are advantageous, depressing core body temperature is not without potential complications. In order to use hypothermia as an ally instead of an adversary in trauma, great care must be exercised to optimize the therapeutic benefits while avoiding the deleterious consequences of unregulated cooling.

#### A. Dysrhythmias

Induction of hypothermia causes an initial increase in heart rate, cardiac output and systemic vascular resistance followed by a decrease in heart rate and cardiac output once temperatures are less than 30°C. Typically, heart rate slows down markedly below 28°C and eventually leads to asystole by the time deep hypothermia is achieved. Since induced hypothermia also reduces metabolic demand, this negative chronotropic effects should not significantly disrupt the energy supply and demand equilibrium. Moderate hypothermia has also been associated with elevated risk of cardiac dysrhythmias, J waves, first degree heart block, and prolonged QT [111]. A clinical trial of moderate hypothermia in pediatric traumatic brain injury patients observed increased arrhythmias (which were manageable with standard interventions) in their hypothermia group and a trial of moderate hypothermia for neonatal encephalopathy reported more frequent bradycardia[112,113]. The generalizability of these findings to adult trauma patients is uncertain. Correspondingly, continuous monitoring of cardiac and metabolic parameters is essential, but potential for complications is small for mild-moderate hypothermia.

#### B. Coagulopathy

Hypothermia decreases the enzymatic activity of clotting factors and reduces the number and function of platelets [114]. This effect is dependent upon the depth of hypothermia and becomes clinically measurable when core temperature drops below 33°C. Thus, it is not surprising that the incidence of coagulopathy is inconsistent in pre-clinical and human studies of mild-moderate hypothermia. Mild hypothermia has been associated with increased PT and PTT in a porcine model of hemorrhagic shock [89] but it had no significant coagulopathic effects in other animal studies [71,115]. In pediatric patients, deep hypothermic circulatory arrest during cardiac surgery has been associated with increased bleeding and transfusion requirements [116]. In adults, a study of trauma patients reported impaired platelet function but normal fibrinolysis [117]. And, a meta-analysis of clinical trials of mild therapeutic hypothermia for traumatic head injury determined that PTT was slightly increased (0.02 seconds) in the hypothermia group [118].

Consequently it is important to closely monitor coagulation parameters. However, coagulopathy does not uniformly occur until the temperature drops below 33°C. Also, in the setting of mild to moderate hypothermia the changes in laboratory measures of coagulation do not necessarily lead to worse outcomes.

Deep and profound hypothermia indeed cause *reversible *coagulopathy which corrects rapidly once the temperature is brought back to normal. The consequences of this coagulopathy must be measured against the potential advantages. Clearly excessive blood loss in a trauma patient is not desirable. But short durations of coagulopathy may be tolerable when controlling life threatening hemorrhage. For these select patients, transient coagulopathy may be an acceptable tradeoff to preserve key organs during periods of critical ischemia. Interestingly, as the tissue oxygen demands are minimal during profound hypothermia, coagulopathy and additional blood loss are very well tolerated in animal models.

#### C. Infections

Hypothermia blunts the immune response, and decreases cytokine production and neutrophil migration. While this immunological attenuation protects tissues from reperfusion injury and inflammatory damage, it may also increase the risk of infection. In a randomized controlled trial of elective surgery patients, normothermia reduced surgical wound infection rates when compared to perioperative mild hypothermia [119]. Studies have also reported increased pneumonia in patients receiving therapeutic hypothermia for traumatic brain injury [120,121]. These patients are clearly at a high risk for developing pneumonia due to need for prolonged mechanical ventilation. At the same time, several studies have shown no increase in the infection rates with the use of hypothermia [112,122].

As with coagulopathy, risks of infections must be balanced against the life saving potential of this therapeutic intervention. The duration and depth of hypothermia are critical variables that influence the infection rates--a short duration of profound hypothermia is not the same as prolonged periods of mild-moderate hypothermia. For example, no infectious complications were noted during a six week observation period in our animal models in which lethal vascular injuries were repaired during a short period (60 minutes) of profound (10°C) hypothermia [123]. However, it is reasonable to start perioperative antibiotic coverage prior to instrumentation and induction of hypothermia, and ventilator-associated pneumonia (VAP) precautions should also be routinely maintained in intubated subjects.

#### D. Drug metabolism

Hypothermia decreases the systemic clearance of cytochrome P450 metabolized drugs between approximately 7% and 22% per degree Celsius below 37°C. The therapeutic index of drugs is narrowed during hypothermia and the pharmacokinetics of many drugs may be altered [124]. Treating physicians should be aware of these changes and adjust pharmacotherapy accordingly.

Table 1 summarizes the major physiologic and metabolic changes that need to be considered in hemorrhagic shock complicated by hypothermia, and in the induction of mild therapeutic hypothermia and emergency preservation and resuscitation (EPR).

**Table 1:** Comparison of physiologic and metabolic changes associated with hemorrhagic shock with those changes specific to hemorrhagic shock combined spontaneous hypothermia or induced therapeutic hypothermia.

	Hemorrhagic Shock	Hemorrhagic Shock Complicated by Spontaneous Hypothermia	Therapeutic Mild Hypothermia in Hemorrhagic Shock	Emergency Preservation Resuscitation in Hemorrhagic Shock
**Cardiac**	Hypotension, tachycardia Hypovolemic shock	Cardiac depression Bradycardia Arrhythmias with worsening hypothermia - J waves	Bradycardia Decreased risk of cardiac arrest	Induced cardiac arrest on CPB with low flow state
**Respiratory**	Variable	Central respiratory depression	Variable Carefully monitored	Patient intubated and monitored on ventilator
**Metabolic**	Increase oxygen requirements Switch from aerobic to anaerobic metabolism	Energy (ATP) depletion Supply/demand mismatch	Reduced metabolic requirements allowing supply to meet demand	ATP reserved with reduced metabolism. No oxygen or substrates requirements
**Coagulopathy**	Trauma-induced consumptive coagulopathy Reperfusion dilutional coagulopathy	Decrease platelets and coagulation factors activity	Continuous monitoring of PT and PTT	Irrelevant
**Mental Status**	Variable	Progressive depression in mental status and eventually coma with flat EEG	Patient deliberately sedated and paralyzed	Patient deliberately sedated and paralyzed
**Immune system**	Initiation of inflammatory response with multiple organ damage	Blunted cytokine production and neutrophil migration with increased risk of infections	Decreased immune and inflammatory response Antibiotic prophylaxis	Antibiotics coverage and sepsis precaution during hypothermia induction
**Shivering**	Not observed early in hemorrhage	Increased attempt to produce heat increases energy demand and over-utilizes ATP	Muscular blockade to control shivering.	Muscular blockade to control shivering
**Hyperglycemia**	Irrelevant	Decreased insulin production and resistance leads to hyperglycemia	Controlled and reversible	Controlled and reversible

ATP = Adenosine Tri-Phosphate. EEG = Electroencephalogram.

### Novel Strategies: Beyond the Golden Hour

Current management strategies are inadequate in the face of advanced hemorrhage. There is a clear need to develop a novel approach that can extend the *Golden Hour*, facilitate surgical repair, modulate metabolic demands, attenuate immune and inflammatory responses, and preserve vital tissues. Pre-clinical data strongly suggests that induced hypothermia has significant potential as a therapeutic modality for Emergency Preservation and Resuscitation (EPR) in severe trauma.

### Therapeutic Hypothermia in Bleeding Trauma Patients: Preclinical Potential

Most studies demonstrating beneficial effects of therapeutic hypothermia have focused on clinical scenarios such as cerebral ischemia, cardiac arrest, and transplantation. Although no human studies have been performed to evaluate its application in the setting of hemorrhagic shock, there are numerous large and small animal studies that address this issue. These pre-clinical data suggest that the optimal protocol for bleeding trauma patients would differ depending on the severity of hemorrhage, presence or absence of additional injuries, and time required for definitive surgical repair. Thus, the published pre-clinical studies have been divided according to severity of hemorrhagic insult to correlate with different clinical scenarios.

#### Hypotensive Bleeding Trauma Patients

Severe bleeding is a frequent cause of hypotension and shock in trauma patients. Since hypotension does not develop until >30-40% blood volume loss, these injuries are often fatal and must be managed rapidly to avoid death. In this patient population, our goal should be to delay the onset of cardiac arrest and create adequate time for surgical repair/control of hemorrhage, as well as provide cellular protection from ischemia/reperfusion injury during fluid resuscitation following surgery. Therapeutic hypothermia is a promising option. Mild to moderate hypothermia decreases heart rate and increases systemic vascular resistance while maintaining stroke volume and blood pressure. Hence, hypothermia decreases cardiac metabolic demands while sustaining cardiac output and myocardial perfusion [97]. In a model of uncontrolled hemorrhage, mild to moderate hypothermia induced by surface cooling delayed the onset of cardiac arrest and significantly improved survival in rats [125-127]. Large animal models confirmed that the induction of hypothermia (varying depths) after hemorrhagic shock can improve survival, and preserve organ viability and cognitive function [128-130]. These benefits were also seen in hemorrhaged animals that underwent repair of multiple concurrent injuries without an increase in postoperative complications [123]. This survival benefit can be further enhanced by coupling cooling with minimal intravenous fluid resuscitation [131].

Investigators have not only observed cardioprotective effects and survival advantage, but also systemic protection through attenuation of the inflammatory response [132]. In a small animal model of lethal hemorrhage treated with profound hypothermia, our team has demonstrated decreased lactic acidosis and improved survival along with a significant decrease in apoptotic proteins, preserved phosphorylated Akt levels, and increased prosurvival intermediates such as Bcl-2 and β-catenin proteins [101]. Similarly, in a swine model of lethal vascular injuries, profound hypothermia modulated the post-shock immune response by attenuating the pro-inflammatory IL-6, increasing anti-inflammatory IL-10 and augmenting the expression of protective HSP-70 protein [133]. Additional studies suggest that hypothermia can protect the brainstem from oxidative stress during hemorrhagic shock by decreasing glutathione levels [134], microvascular permeability and generation of ROS [135].

The optimal time course and depth of hypothermia in hypotensive trauma patients remains unknown. Preclinical studies indicate that mild hypothermia may be preferable to deeper cooling. This strategy would provide adequate protection while minimizing the cardiac, coagulation and immunological complications observed at lower temperatures [136]. Moreover, the ability to induce a protective hypothermic state with surface cooling makes this a suitable strategy in resource poor settings (e.g. pre-hospital, battlefield). With regard to duration, animal studies suggest that cooling should be initiated rapidly upon arrival and maintained during surgical repair and reperfusion, and then reversed actively once hemodynamic control has been reestablished. There is also evidence to suggest that extending mild therapeutic hypothermia beyond the early resuscitation period provides no additional benefits. In that study, rats that became spontaneously hypothermic during hemorrhagic shock demonstrated no long term survival advantage when maintained in a mildly hypothermic state for 2 or 12 hours post-resuscitation [115]. In fact, restoration of normothermia during resuscitation seems to improve cardiac and hepatic circulation and function more effectively [132].

#### Bleeding Trauma Patient in Full Cardiac Arrest

Hemorrhage that progresses to full arrest has dismal prognosis. Survival is unlikely unless the source of bleeding can be controlled within minutes and the damage endured during cardiovascular collapse and reperfusion minimized [137]. Emergency department thoracotomies and lengthy resuscitation efforts often fail to improve outcomes with overall survival rates of approximately 7% [138,139]. Nonetheless, a notable portion of these patients die with injuries that could have been repaired, if the surgeons were afforded some additional time to do so. In this patient population, the most promising intervention in preclinical studies has been the rapid induction of profound hypothermia. This strategy, termed *emergency preservation and resuscitation *(EPR) is a period of drastically decreased metabolism to preserve organs and allow surgical repair, followed by controlled resuscitation. EPR aims to extend the *Golden Hour *and preserve organ viability using a profoundly hypothermic state, upregulate pro-survival genes and proteins, and attenuate inflammation [140-142].

Through several rigorous experiments, Dr. Peter Safar and his team at the University of Pittsburgh established the effectiveness of 'suspended animation' strategy in large animal models [143]. These studies determined that cooling to temperatures of 10-15°C were optimal for preservation of severely injured animals that simulated trauma patients in cardiac arrest. Following pressure-controlled hemorrhagic shock (mean arterial pressure 40 mmHg), induction of profound hypothermia and cardiac arrest via closed-chest cardiopulmonary bypass (CPB) for 60 minutes yielded 100% survival in a canine model. EPR's effectiveness following a period of shock makes it very attractive in a civilian or military setting where patients must survive significant transport times before induction of hypothermia (and surgical repair) is possible.

Profound hypothermia can also be successfully induced with high-volume saline infusion into the aorta via a femoral artery catheter [144,145]. The Pittsburgh group achieved 100% survival without significant brain damage for cardiac arrests lasting as long as 90 minutes. Other studies using controlled methods of blood exchange, (removing blood after induction of hypothermia) or after pressure controlled hemorrhage have achieved similar results [146,147]. Profound hypothermia time has been extended to 3 hours using organ-preservation fluids and low-flow CPB during cardiac arrest [148].

### Optimal Technique and Future Directions: Moving from Bench to Bedside

These exciting early EPR experiments were not without limitations. All of the early EPR studies used volume and pressure controlled hemorrhage models without any associated injuries, which incompletely represents the polytrauma patients. In fact, when a mild splenic injury was added to the standard pressure controlled hemorrhagic shock (60 minutes) model, EPR's protective effects were much less impressive, with fifty percent of the animals exhibiting neurological deficits (without histological damage) [149].

Furthermore, several of the methods of hypothermic induction used in these EPR studies are not clinically realistic in trauma setting. Even though an aortic flush via femoral cut-down and the use of CPB are very effective methods, this requires large amounts of fluids to reach the desired degree of cooling. Obtaining femoral access in a pulseless patient presents an added challenge. Therefore, an emergent thoracotomy with direct aortic cannulation and the use of closed loop CBP with a low flow state using acellular fluids during the period of hypothermia could be more practical for rapid induction and maintenance of profound hypothermia [150].

To close the gap between laboratory experiments and clinical reality, our group developed new models that closely mimic the bleeding, polytrauma patients that need treatment in modern trauma medicine. These models used large animals with multiple vascular injuries causing uncontrolled hemorrhage coupled with multiple soft tissue injuries. In this way, we generated a state of "irreversible shock": a highly lethal injury that is unresponsive to traditional resuscitation with crystalloid, blood products and open cardiac massage. However, this injury can be treated successfully with EPR using profound hypothermia and controlled resuscitation on cardiopulmonary bypass [151].

We induced up to 90 minutes of profound hypothermia via an emergency thoracotomy approach to facilitate total body preservation during surgical repair and resuscitation. Long-term survival was excellent without any neurological damage or significant organ dysfunction [151]. The follow up study [127] demonstrated 75% long-term survival could be achieved with one hour of normothermic shock simulating transport time followed by 60 minutes of hypothermic cardiac arrest and repair of lethal vascular injuries above and below the diaphragm. In multiple experiments using this model, all of the normothermic control animals died, whereas almost 90% of those undergoing profound hypothermia survived neurologically intact and with normal cardiac function.

Subsequent studies have outlined that maximum benefits are achieved when profound hypothermia is induced rapidly (2°C/min) and reversed more slowly (0.5°C/min). Optimal depth has also been shown to be 10°C with decreased survival demonstrated at ultra profound temperature (5°C) [152]. In our experiments, rewarming was active and begun promptly after hemorrhage was controlled using cardiopulmonary bypass with the use of blood products as needed to reverse coagulopathy and restore normal oxygen delivery. We delayed blood transfusion until after injuries were repaired and metabolic demands were increased in the re-warming period. Using this optimized model, 60 min of profound hypothermia achieved 90% survival in a model that is 100% lethal in normothermic animals.

Uniquely, coagulopathy and intraoperative bleeding are not a major problem during profound hypothermia since tissue viability is independent of oxygen delivery. Profound hypothermia renders coagulopathy and bleeding irrelevant. Shed blood could be recycled and any hypothermia-induced coagulopathies were completely reversed upon re-warming. Even in the presence of vascular, splenic, and colonic injuries, we have successfully induced profound hypothermia without a significant increase in post-operative bleeding or infection [120]. We also tested a portable, battery-operated, rotary pump to induce hypothermia in large animal model. This portable device performed as well as the conventional roller pump, and it seems to be more suitable in austere setting and pre-hospital environment during transport [153].

Using this optimal approach that synthesizes emergency preservation and resuscitation (EPR), we believe that irreversible severe hemorrhagic shock presenting with cardiac arrest can be successfully reversed. Contemporary EPR strategies using a flush solution supplemented with oxygen and glucose can prolong life for as long as 180 minutes with adequate neurological recovery following pressure controlled hemorrhage [154]. Many questions remain and improved life-preserving strategies desired. What is the maximum duration for EPR in the care of bleeding, polytrauma patients? Since hypothermia does not completely abolish metabolic activity, could EPR be augmented by alternative resuscitation fluids or cell preserving therapies [155]?

## Conclusions

From our extensive pre-clinical experience and review of the current literature, we contend that induced hypothermia can protect tissues and improve survival in bleeding polytrauma patients. Moreover, EPR strategies can effectively prolong survivable ischemia time in pulseless trauma patients and buy additional time for surgical repair of exsanguinating injuries followed by early fluid resuscitation. Significant advances have been made in our understanding of the physiological, inflammatory, and cell biological changes that facilitate the protective effects of therapeutic hypothermia and the deleterious consequences of spontaneous hypothermia. Thus, although it is important to acknowledge that hypothermia is a double edge sword, our ability to make significant strives in clinically-relevant experiments should encourage us to bridge the gap between bench and bedside. As the mortality rate from hemorrhagic shock continues to rise, it is incontrovertible that further research, in the form of a rigorous, controlled clinical trial, must be done to translate these promising findings into trauma practice.

## Competing interests

The authors declare that they have no competing interests.

## Authors' contributions

HA conceived the project and provided the overall structure of the manuscript. TK and AK performed the literature review and composed the manuscript. All of authors read and approved the final manuscript.
